# Cognitive Functions and Depression in Patients with Irritable Bowel Syndrome

**DOI:** 10.1155/2015/438329

**Published:** 2015-05-21

**Authors:** Per G. Farup, Knut Hestad

**Affiliations:** ^1^Department of Research, Innlandet Hospital Trust, 2380 Brumunddal, Norway; ^2^Unit for Applied Clinical Research, Department of Cancer Research and Molecular Medicine, Faculty of Medicine, Norwegian University of Science and Technology, 7491 Trondheim, Norway; ^3^Faculty of Social Sciences and Technology Management, Norwegian University of Science and Technology, 7491 Trondheim, Norway; ^4^Hedmark University College, 2409 Elverum, Norway

## Abstract

*Background*. Irritable bowel syndrome (IBS) is associated with depression and depression with impaired cognitive functions. The primary aim was to study associations between depression and cognitive functions in patients with IBS. *Methods*. IBS (according to the Rome III criteria), cognitive functions (evaluated with a set of neuropsychological tests), and depression (measured with Beck Depression Inventory II and Montgomery-Åsberg Depression Scale) were analysed in patients with idiopathic depression and in patients with unspecified neurological symptoms. *Results*. 18 and 48 patients with a mean age of 47 and 45 years were included in the “Depression” and “Neurological” group, respectively. In the “Depression” group, the degree of depression was significantly higher in patients with IBS than in those without. Depression was associated with impaired cognitive function in 6 out of 17 neuropsychological tests indicating reduced set shifting, verbal fluency, attention, and psychomotor speed. IBS was statistically significantly associated with depression but not with any of the tests for cognitive functions. *Conclusions*. IBS was associated with depression but not with impaired cognitive functions. Since the idiopathic depression was associated with cognitive deficits, the findings could indicate that the depression in patients with IBS differs from an idiopathic depression.

## 1. Introduction

Irritable bowel syndrome (IBS) is a common disorder with a high prevalence of comorbidities such as musculoskeletal pain, anxiety and depression, and emotional disturbances and is common in patients with an idiopathic depression [1–4]. The two disorders, IBS and the idiopathic depression, are associated and have in common several pathophysiological abnormalities [5, 6]. The interaction between the gut and the brain called the “brain-gut axis” is mediated via humoral, immunological, and neuronal pathways. The axis is of importance for health and disease including gastrointestinal and psychological functions [7].

Cognitive deficits are common in patients with an idiopathic depression but have been less well studied in patients with IBS. The associations between IBS and cognitive functions are contradictory in part due to different methods for evaluation of the cognition [8–13]. The main reason for this study was the contradictory information about cognitive functions in patients with IBS, particularly the association between depression and cognitive functions. Knowledge about the association between depression and cognitive functions in patients with IBS is important for a better understanding of the “brain-gut axis” and a correct evaluation of the patients. Kennedy et al. pinpoint the lack of knowledge very well:* “…it will be necessary to carefully control for psychiatric co-morbidity so that the relative contributions of anxiety and depression to deficits in cognitive functioning can be disentangled from the alterations associated with IBS alone”* [14].

This study was designed to compare patients with an idiopathic depression and unspecified neurological symptoms. The protocol specified the supplementary analyses related to IBS. The primary aim of these analyses was to study the associations between depression and cognitive functions in patients with IBS.

## 2. Methods

### 2.1. Design and Participants

#### 2.1.1. Design

Design was cross-sectional studies in two groups of patients.

- Consecutive patients above 17 years of age with the diagnosis of idiopathic depression (according to ICD-10; F 32–34 spectre, without triggering factors) referred to a psychiatric outpatient clinic were included in the study after exclusion of organic diseases (the “Depression” group).
- Consecutive patients above 17 years of age admitted to an inpatient neurological clinic for thorough investigations of neurological symptoms were included after exclusion of organic disorders (the “Neurological” group). The patients had no objective neurological signs, all laboratory tests were normal, and all supplementary investigations (CT, MRI, spinal fluid examination, etc.) which were performed at the clinicians' discretion were normal. The patients presented with various symptoms such as headache, back pain, and vertigo.

A medical history was recorded, a routine clinical examination was performed, and haematological and biochemical screening tests were taken in all patients. In order to exclude other diseases, other tests were accomplished according to the doctors' discretion. All patients filled in validated questionnaires for the classification of gastrointestinal disorders and depression. A set of neuropsychological tests was carried out. An experienced psychiatric study nurse performed the practical work with the questionnaires and the neuropsychological testing.

### 2.2. Variables

The following variables were used.

#### 2.2.1. Demographics

The demographics were the following.

- Gender; age (years); education (number of years in school).

#### 2.2.2. Abdominal Complaints

The abdominal complaints were assessed as follows.

- IBS (yes/no) was assessed with a validated Norwegian version of the internationally accepted Rome III questionnaire [15, 16].
- The degree of abdominal symptoms was measured with Irritable Bowel Severity Scoring System (IBSSS) (mild 75–174; moderate 175–300; and severe 301–500) [15, 16].

#### 2.2.3. Depression

Two valid and reliable questionnaires were used for the scoring of depression.

- Beck Depression Inventory v. II (BDI-II): minimal depression 0–13; mild depression 14–19; moderate depression 20–28; and severe depression 29–63 [17].
- Montgomery-Åsberg Depression Scale (MADRS): normal/symptom absent 0–6; mild depression 7–19; moderate depression 20–34; and severe depression 35–60 [18].

#### 2.2.4. Cognitive Function

Several valid and reliable tests were used for the evaluation of cognitive functions [19]. The tests evaluate different aspects of the cognitive functions.

- Mini-Mental State Examination (MMSE) (score 0–30, normal ≥ 24) is a test for dementia [20].
- Trail Making Test A (simple) (ref. value 30 (SD 10) seconds); Trail Making Test B (complex) (ref. value 60 (SD 15) seconds): the Trail Making Test measures attention, visual searching, mental processing speed, set shifting, and cognitive flexibility. Low scores are best.
- Grooved Pegboard Test is a test of fine motor control. The test measures time in seconds with dominant hand (ref. value 63 (SD 10) seconds) and nondominant hand (ref. value 69 (SD 15) seconds). Low scores are best.
- Hopkins Verbal Learning Test (HVLT) immediate total recall (score 0–36) and HVLT delayed recall (score 0–12) are tests of verbal immediate and delayed recall. High scores are best.
- Brief Visual Memory Test (BVMT) immediate total recall (score 0–36) and BVMT delayed recall (score 0–12) measure immediate and delayed visual recall. Both tests measure the number of words or items the participant can recall. High scores are best.
- Wechsler Adult Intelligence Scale 3rd Edition (WAIS-III) Vocabulary (ref. value raw score 46) measures the patients' ability to define and explain different words. WAIS-III Digit Symbol (ref. value raw score 64) and WAIS-III Symbol Search (ref. value raw score 26) measure processing speed, visual perception, attention and concentration, and motor and mental speed (number of correct answers within a period) [21, 22].
- Stroop Test 1 (Word) (colour naming) (ref. value 41, SD 7); Stroop Test 2 (Colour) (colour name reading) (ref. value 53, SD 9); Stroop Test 3 (Interference) (colour interference) (ref. value 85, SD 18) [23]: the Stroop tests measure attention, cognitive processing, and mental stimulus control. The results are given as time in seconds. Low scores are best.
- Controlled Oral Word Association Test (COWAT) (Words) (number of Words on F, A, and S) (ref. value 31, SD 5); COWAT (Cloths) (number of cloths) (ref. value 13, SD 3); COWAT (Animals) (number of animals) (ref. value 17, SD 5) [24–26]: these are all verbal fluency tests that include measures of verbal, cloths, and animal material. The tests measure the ability to generate words beginning with a given letter or category within one minute. High scores are best.

### 2.3. Statistics

The characteristics of the patients were analysed with descriptive statistics and reported as mean (SD), median (range), and proportion (percentage). Comparisons between the groups were analysed with an exact unconditional test for 2 × 2 tables, *t*-test, and Mann-Whitney *U* test depending on the type of the data and the normality. Predictors of depression and cognitive functions were studied with univariate general linear model analyses with the scores for depression and cognitive functions (one at a time) as dependent variables. Independent variables were the groups “Neurological”/“Depression,” “no IBS”/“IBS,” gender (fixed factors), and age and education (covariates). For the calculation of estimated marginal means, the interaction between the groups “Neurological”/“Depression” and “IBS”/“no IBS” was added to the model. Except for the adjustments made in the multivariable analyses, no adjustments were made for multiple comparisons.

## 3. Results

### 3.1. Patients

Out of 71 patients included in the study for comparisons between patients in the “Neurological” and “Depression” group, 5 in the “Neurological” group were excluded because of organic abdominal diseases or incompletely filled-in questionnaires. This left 66 patients for the analyses: 18 in the “Neurological” group and 48 in the “Depression” group.

### 3.2. Descriptive and Univariable Analyses

Table 1 gives the characteristics of the participants with comparisons between the groups. Compared to the patients in the “Neurological” group, the patients in the “Depression” group had statistically significantly higher scores for depression and abdominal complaints (IBSSS), significantly impaired cognitive functions on 5 out of 17 tests, and a trend for a higher prevalence of IBS. Attention, cognitive processing, and verbal fluency were the cognitive functions with the most marked differences between the groups. Tables 2 and 3 compare patients with and without IBS in the two groups. The cognitive functions did not differ significantly between the patients with and without IBS in any of the two groups. In the “Depression” group, depression and abdominal complaints were significantly more severe in patients with IBS.

### 3.3. Univariate Regression Analyses

**Figure 1:**
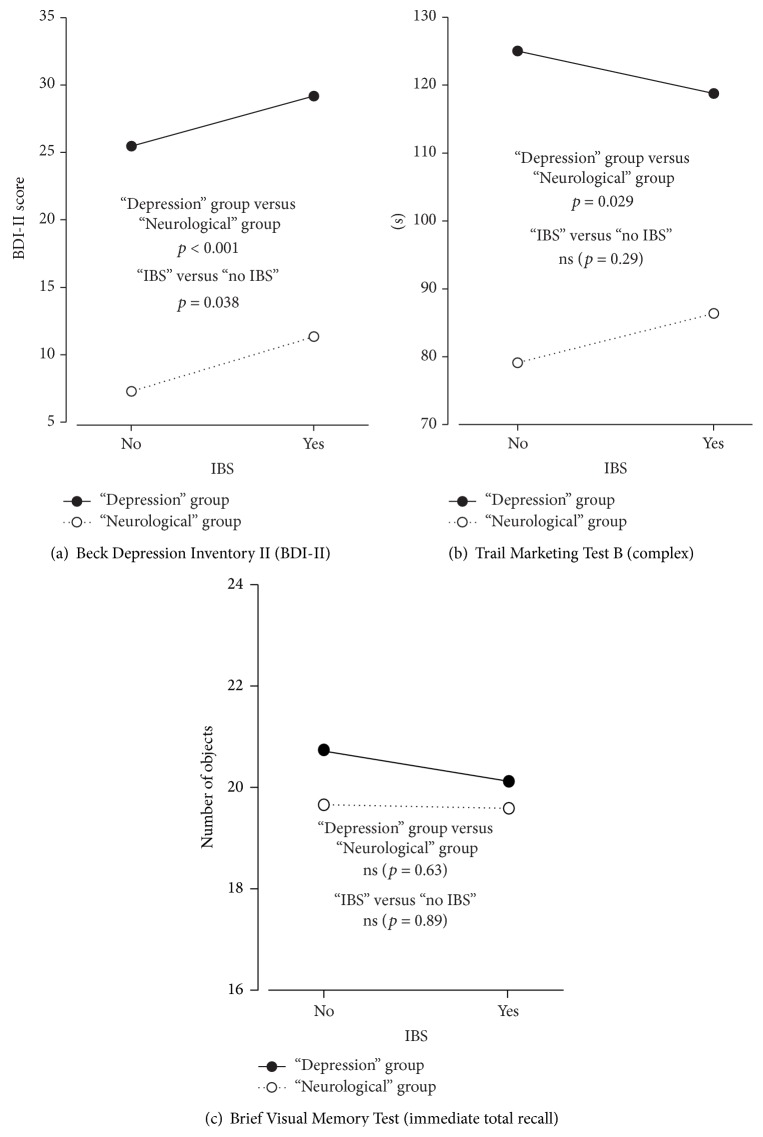
Estimated marginal mean scores for Beck Depression Inventory II (a), Trail Making Test B (b), and Brief Visual Memory Test immediate total recall (c) in the “Depression” and “Neurological” groups divided into patients with and without IBS after adjusting for age, sex, and education.

IBS was an independent predictor of depression but was not associated with differences in any of the tests for cognitive functions. The “Depression” group was associated with significantly reduced cognitive performance in 6 of the 17 tests. The most marked differences were seen in the tests for attention and cognitive processing (Stroop 1 (Word)) and verbal fluency (COWAT (Animal)).  Table 4 gives the details.  Figure 1 visualises the associations between the groups with and without IBS and the “Neurological” and “Depression” groups for some selected variables (BDI-II, Trail Making Test B, and HVLT immediate total recall). Both the “IBS” group and the “Depression” group were significantly associated with BDI-II, only the “Depression” group was associated with Trail Making Test B, and none of them was associated with HVLT immediate total recall.

## 4. Discussion

The main finding was that the cognitive functions measured with a broad spectre of reliable and validated tests were unrelated to IBS. In accordance with other studies, this study showed associations between IBS and depression and between the idiopathic depression and cognitive functions. We are not aware of previous studies on the association between IBS and cognitive functions after adjusting for the idiopathic depression. These new findings are of importance for the clinical evaluation of patients with IBS. Since depression and other comorbidities are common in patients with IBS, physicians might wrongly suspect those with depression or cognitive impairment.

The normal cognitive functioning in patients with IBS has, with some exceptions, also been reported in other studies [9, 10, 14]. Kennedy et al. used several tests including Paired Associates Learning (PAL) test. They reported a subtle visuospatial memory deficit that remained after correction for psychiatric comorbidity, in one out of 5 PAL subtests, but no change in any of the other tests [10]. Brain imaging (functional magnetic resonance imaging and positron emission tomography) and neurophysiological recordings (cerebral evoked potentials, magnetoencephalography, and spinal reflex responses) have shown abnormal findings in patients with IBS. The clinical relevance of these findings, such as the relation to affective and cognitive functions, has not been established [27, 28].

The results have theoretical and practical implications. Theoretically, impaired cognitive performance was expected since patients with IBS are often depressed and patients with depression have impaired cognitive performance. The aetiology, pathogenesis, and pathophysiology of depression are complex and might differ between various forms of depression such as “idiopathic depression,” “reactive depression,” and “inflammation associated depression” [29–31]. Depression might be several diseases or disorders with unequal associations with cognitive functioning and different associations with the brain-gut axis.

The findings are also of importance for clinical practice. Patients with IBS are sometimes regarded as “nagging” persons since they present with a wide range of comorbidities including anxiety, depression, muscle-skeletal pain, unexplained somatic symptoms, and poor social functioning. This study showed that their cognitive and intellectual functions were unaffected and indirectly indicates that their comorbidity is “real.” The findings should remind the clinicians not to assign patients with IBS of more comorbidities than necessary and to handle the symptoms they present seriously.

The tight association between IBS and depression shown in this study is well known from other studies, as is the association between depression and cognitive performance [1–3, 8, 32–34]. This study showed that the cognitive functions in patients with depression were unequally affected. A significant impairment was related to the visual scanning, motor speed, and set shifting (the Trail Making Tests and the Stroop Tests) and to fine motor control and tempo (Grooved Pegboard Tests). The capacity for immediate and delayed recall (the HVLT and BVMT) was unaffected. The impaired WAIS-III Digit Symbol test, which has been evaluated as one of the most sensitive WAIS-III tests, could indicate intellectual impairment. Other functional differences were less clear. Overall, the results indicate that the set shifting, verbal fluency, attention, and psychomotor speed were reduced in patients with depression, whereas other functions were normal.

The gut and the brain interact through a bidirectional neuronal, humoral, and immunological communication referred to as the brain-gut axis that affects both gastrointestinal and psychological functioning [7]. The system is only partly understood, but the influence of the gut microbiota and the function of the blood-brain barrier on the system have been ascertained [35, 36]. Both IBS and depression are influenced by the brain-gut axis and have some common pathophysiological abnormalities that could explain the associations between the two disorders [5, 6, 31, 37]. The importance of the brain-gut axis for cognitive functioning is unknown. The finding that there were no associations between the gut and cognitive functions could indicate that the interaction between the gut and depression differs from the interaction between the gut and cognitive functions.

### 4.1. Strengths and Limitations

The use of a wide range of valid and reliable neuropsychological tools for the evaluation of cognitive functions is a significant strength of this study. Some other studies have used tool measuring psycho-social-emotional-thinking and not strict neuropsychological functioning that could explain the contradictory results [12, 13].

The case-control design of the study was planned for comparisons between patients with and without depression and was not ideal for the study of IBS. Nevertheless, the design made the planned comparisons between patients with and without IBS in the two groups possible, and the analyses were according to the protocol. The “Neurological” group was used as controls because no somatic or psychiatric disorder could explain their unspecific neurological symptoms. A completely healthy control group would have been preferable. In addition to their unspecific neurological symptoms, the “Neurological” group had a high prevalence of comorbidities such as IBS and abdominal complaints and perhaps affective and cognitive disorders.

IBSSS has been validated for the scoring of symptoms in subjects with IBS and not for the scoring of all functional gastrointestinal disorders, as it was used in this study. This use of IBSSS makes the results of the IBSSS less reliable and explains the high scores in patients without IBS.

The lack of any significant differences between patients with and without IBS in the “Neurological” group was probably a type II error due to the small sample size. Not even the IBSSS differed between the groups, which indicated a high prevalence of gastrointestinal symptoms also in subjects without IBS.

There was no tendency toward cognitive deficits in patients with IBS despite having significantly more depression. The total sample size was limited, and a type II error cannot be excluded. If an association between IBS and cognitive deficits has been missed, the association must be weaker than between IBS and depression, which was highly significant.

## 5. Conclusions

There were no significant associations between IBS and cognitive functions. IBS was associated with depression, and the idiopathic depression was associated with cognitive deficits. The findings could indicate that depression in patients with IBS differs from an idiopathic depression and that the interaction between the gut and depression differs from that of the gut and cognitive functions.

## Figures and Tables

**Table 1:** Patient characteristics and comparisons between the “Neurological” and “Depression” groups.

Patient characteristics	“Neurological”	“Depression”	Statistics
Gender (female/male)	12 (63%)/7 (37%)	25 (53%)/22 (47%)	*p* = 0.59
Age (years)	47.3 (14.5)	44.7 (14.0)	*p* = 0.51
Education (years)	13 (10–17)	13 (10–17)	*p* = 0.15^∗^
IBS	6 (32%)	27 (57%)	*p* = 0.07
IBSSS	113 (88)	200 (103)	**p** = 0.003
BDI-II	5 (0–26)	29 (10–54)	**p** < 0.001^∗^
MADRS	8.1 (5.9)	27.5 (8.1)	**p** < 0.001
MMSE	29 (27–30)	29 (23–30)	*p* = 0.12^∗^
Trail Making A (simple)	34 (20–145)	45 (22–153)	**p** = 0.01^∗^
Trail Making B (complex)	71 (45–181)	88 (49–361)	**p** = 0.02^∗^
Grooved Pegboard Test (dominant hand)	70 (54–112)	78 (53–176)	*p* = 0.14^∗^
Grooved Pegboard Test (not dominant hand)	74 (55–135)	87 (61–228)	**p** = 0.02^∗^
HVLT immediate total recall	26 (10–29)	23 (11–34)	*p* = 0.29^∗^
HVLT delayed recall	10 (1–12)	9 (10–12)	*p* = 0.37^∗^
BVMT immediate total recall	20.7 (5.4)	19.7 (6.9)	*p* = 0.51
BVMT delayed recall	8.6 (2.3)	7.7 (2.5)	*p* = 0.19
WAIS-III Vocabulary	43.0 (6.7)	44.0 (8.5)	*p* = 0.65
WAIS-III Digit Symbol	60.3 (15.1)	51.8 (16.9)	*p* = 0.06
WAIS-III Symbol Search	28.1 (8.7)	25.0 (8.2)	*p* = 0.19
Stroop 1 (Word)	32 (25–45)	38 (25–67)	**p** = 0.003^∗^
Stroop 2 (Colour)	26 (20–39)	29.0 (19–52)	*p* = 0.17^∗^
Stroop 3 (Interference)	54 (39–118)	61 (43–141)	*p* = 0.36^∗^
COWAT (Words on F, A, and S)	27 (18–75)	33 (13–69)	*p* = 0.31^∗^
COWAT (Cloths)	19.4 (4.9)	17.6 (5.1)	*p* = 0.21
COWAT (Animals)	22.8 (6.2)	18.6 (5.4)	**p** = 0.008

The results are given as the number (proportion in per cent), mean (SD), and median (range) and analysed with exact unconditional table analyses, *t*-test, and Mann-Whitney *U* test (marked with ∗).

**Table 2:** Patient characteristics and comparisons of patients with and without IBS in the “Neurological” group.

Patient characteristics	No IBS	IBS	Statistics
Gender (female/male)	8 (62%)/5 (38%)	4 (67%)/2 (33%)	*p* = 0.85
Age (years)	46.5 (15.6)	49.2 (12.7)	*p* = 0.72
Education (years)	13 (10–17)	13.5 (10–17)	*p* = 0.90^∗^
IBSSS	87.9 (67.8)	164.5 (107.9)	*p* = 0.08
BDI-II	5 (0–26)	4.5 (2–19)	*p* = 0.90^∗^
MADRS	6.9 (5.0)	10.5 (7.5)	*p* = 0.23
MMSE	30 (28–30)	28.5 (27–30)	*p* = 0.24^∗^
Trail Making A (simple)	32 (20–67)	36 (24–145)	*p* = 0.52^∗^
Trail Making B (complex)	71 (45–130)	69.5 (61–181)	*p* = 0.83^∗^
Grooved Pegboard Test (dominant hand)	70 (54–94)	68 (56–112)	*p* = 0.90^∗^
Grooved Pegboard Test (not dominant hand)	73 (55–131)	75 (62–135)	*p* = 0.64^∗^
HVLT immediate total recall	23 (10–29)	26.5 (20–29)	*p* = 0.28^∗^
HVLT delayed recall	10 (1–12)	10 (6–11)	*p* = 0.58^∗^
BVMT immediate total recall	21.0 (6.0)	20.2 (4.1)	*p* = 0.76
BVMT delayed recall	8.4 (2.6)	9.1 (1.6)	*p* = 0.51
WAIS-III Vocabulary	41.7 (6.6)	45.8 (6.3)	*p* = 0.22
WAIS-III Digit Symbol	62.8 (16.3)	54.7 (11.7)	*p* = 0.29
WAIS-III Symbol Search	29.8 (8.1)	24.3 (9.5)	*p* = 0.21
Stroop 1 (Word)	31 (25–44)	33 (30–45)	*p* = 0.15^∗^
Stroop 2 (Colour)	24 (20–39)	26.5 (22–28)	*p* = 0.42^∗^
Stroop 3 (Interference)	54 (39–118)	58 (48–104)	*p* = 0.77^∗^
COWAT (words on F, A, and S)	31 (18–75)	25 (22–34)	*p* = 0.24^∗^
COWAT (Cloths)	19.7 (5.5)	18.5 (3.6)	*p* = 0.62
COWAT (Animals)	23.2 (7.4)	22.2 (2.0)	*p* = 0.76

The results are given as the number (proportion in per cent), mean (SD), and median (range) and analysed with exact unconditional table analyses, *t*-test, and Mann-Whitney *U* test (marked with ∗).

**Table 3:** Patient characteristics and comparisons of patients with and without IBS in the “Depression” group.

Patient characteristics	“No IBS”	“IBS”	Statistics
Gender (female/male)	12 (60%)/8 (40%)	13 (48%)/14 (52%)	*p* = 0.47
Age (years)	46.3 (16.1)	43.6 (12.4)	*p* = 0.52
Education (years)	12 (7–17)	13 (9–17)	*p* = 0.42^∗^
IBSSS	159 (109)	230 (89)	**p** = 0.02
BDI-II	22.5 (10–49)	37 (10–54)	**p** = 0.007^∗^
MADRS	24.7 (7.3)	29.5 (8.2)	**p** = 0.04
MMSE	29 (23–30)	29 (25–30)	*p* = 0.34^∗^
Trail Making A (simple)	44.5 (22–113)	45 (24–153)	*p* = 0.95^∗^
Trail Making B (complex)	88.5 (49–361)	88 (50–361)	*p* = 0.85^∗^
Grooved Pegboard Test (dominant hand)	83.5 (53–176)	76 (54–163)	*p* = 0.36^∗^
Grooved Pegboard Test (not dominant hand)	89.0 (61–172)	87 (63–228)	*p* = 0.48^∗^
HVLT immediate total recall	23.5 (11–29)	23.0 (15–34)	*p* = 0.81^∗^
HVLT delayed recall	9 (1–12)	9 (5–12)	*p* = 0.74^∗^
BVMT immediate total recall	19.5 (6.5)	19.9 (7.3)	*p* = 0.85
BVMT delayed recall	8.0 (2.3)	7.6 (2.7)	*p* = 0.64
WAIS-III Vocabulary	42.5 (8.7)	45.1 (8.3)	*p* = 0.31
WAIS-III Digit Symbol	52.6 (21.1)	51.1 (13.2)	*p* = 0.80
WAIS-III Symbol Search	24.0 (8.6)	25.9 (8.0)	*p* = 0.44
Stroop 1 (Word)	38 (29–56)	38 (25–67)	*p* = 1.00^∗^
Stroop 2 (Colour)	28.5 (19–45)	29 (19–52)	*p* = 0.96^∗^
Stroop 3 (Interference)	67 (43–141)	57 (44–127)	*p* = 0.43^∗^
COWAT (Words on F, A, and S)	33 (13–54)	32 (18–69)	*p* = 0.68^∗^
COWAT (Cloths)	17.9 (5.7)	17.4 (4.6)	*p* = 0.75
COWAT (Animals)	18.2 (5.2)	19.0 (5.7)	*p* = 0.64

The results are given as the number (proportion in per cent), mean (SD), and median (range) and analysed with exact unconditional table analyses, *t*-test, and Mann-Whitney *U* test (marked with ∗).

**Table 4:** The associations between depression and cognitive functions and the groups “Neurological”/“Depression” and “no IBS”/“IBS.” The results of linear regression analyses after adjusting for age, sex, and years of education.

Dependent variables	Independent variables	
	“Neurological”/“Depression” *B* (95% CI); *p* value	“No IBS”/“IBS” *B* (95% CI); *p* value
BDI-II	22.0 (16.0 : 28.0); **p** < 0.001	7.3 (1.9 : 12.7); **p** = 0.009
MADRS	18.1 (14.1 : 22.1); **p** < 0.001	3.8 (0.2 : 7.4); **p** = 0.038
MMSE	−0.6 (−1.5 : 0.3); *p* = 0.19	0.0 (−0.8 : 0.8); *p* = 0.94
Trail Making Test A (simple)	11.0 (−3.6 : 25.7); *p* = 0.14	7.0 (−6.1 : 20.1); *p* = 0.29
Trail Making Test B (complex)	40.7 (4.2 : 77.2); **p** = 0.029	−2.6 (−35.3 : 30.1); *p* = 0.87
Grooved Pegboard Test (dominant hand)	15.7 (3.3 : 28.0); **p** = 0.014	−6.9 (−18.0 : 4.1); *p* = 0.22
Grooved Pegboard Test (not dominant hand)	19.9 (3.6 : 36.1); **p** = 0.017	−5.1 (−19.7 : 9.4); *p* = 0.48
HVLT immediate total recall	−1.3 (−3.9 : 1.3); *p* = 0.33	1.3 (−1.0 : 3.7); *p* = 0.26
HVLT delayed recall	−0.5 (−1.9 : 0.8); *p* = 0.41	0.2 (−0.9 : 1.4); *p* = 0.69
BVMT immediate total recall	−0.9 (−4.3 : 2.6); *p* = 0.63	−0.2 (−3.3 : 2.9); *p* = 0.89
BVMT delayed recall	−1.1 (−2.4 : 0.2); *p* = 0.11	−0.1 (−1.3 : 1.1); *p* = 0.86
WAIS-III Vocabulary	2.3 (−1.4 : 6.1); *p* = 0.22	2.4 (−1.0 : 5.8); *p* = 0.16
WAIS-III Digit Symbol	−8.5 (−15.9 : −1.2); **p** = 0.024	−3.4 (−10.0 : 3.3); *p* = 0.31
WAIS-III Symbol Search	−3.5 (−7.1 : 0.1); *p* = 0.056	−0.5 (−3.8 : 2.7); *p* = 0.74
Stroop 1 (Word)	5.6 (1.6 : 9.7); **p** = 0.007	1.0 (−2.6 : 4.6); *p* = 0.57
Stroop 2 (Colour)	2.2 (−1.2 : 5.7); *p* = 0.19	0.4 (−2.7 : 3.4); *p* = 0.82
Stroop 3 (Interference)	9.5 (−1.5 : 20.6); *p* = 0.09	−3.9 (−13.8 : 6.0); *p* = 0.44
COWAT (Words on F, A, and S)	3.4 (−4.2 : 10.9); *p* = 0.37	−0.1 (−6.9 : 6.6); *p* = 0.97
COWAT (Cloths)	−1.2 (−3.8 : 1.3); *p* = 0.34	−0.6 (−2.9 : 1.6); *p* = 0.58
COWAT (Animals)	−4.5 (−7.6 : −1.4); **p** = 0.005	0.3 (−2.4 : 3.1); *p* = 0.82
